# Hyperbaric oxygen therapy reduces renal lactate production

**DOI:** 10.14814/phy2.13217

**Published:** 2017-03-29

**Authors:** Thomas S. Nørlinger, Per Mose Nielsen, Haiyun Qi, Emmeli Mikkelsen, Kasper Hansen, Nikolaj H. Schmidt, Michael Pedersen, Peter Agger, Fredrik Palm, Christoffer Laustsen

**Affiliations:** ^1^ Department of Clinical MedicineMR Research CentreAarhus UniversityAarhusDenmark; ^2^ Department of Clinical MedicineComparative Medicine LabAarhus UniversityAarhusDenmark; ^3^ Department of PediatricsAarhus University HospitalAarhusDenmark; ^4^ Department of Medical Cell BiologyUppsala UniversityUppsalaSweden

**Keywords:** Hyperpolarization, kidney, kidney metabolism, MRI, type 1 diabetes

## Abstract

Intrarenal hypoxia is an acknowledged factor contributing to the development of diabetic nephropathy. Hyperbaric oxygen (HBO) therapy is a well‐known adjuvant treatment for several medical conditions, such as decompression sickness, infections, and wound healing. The underlying metabolic response of HBO is largely unknown. In this study, we investigated the effect of HBO on the intrarenal metabolic alteration in diabetes. Hyperpolarized [1‐^13^C]pyruvate MRI was performed to assess intrarenal energy metabolism in normoglycemic controls and short‐term (2 weeks) streptozotocin‐induced diabetic rats with and without HBO for five consecutive days. HBO therapy blunted intrarenal lactate production, 3 days after the therapy, in both normoglycemic controls and diabetic rats without affecting either lactate dehydrogenase mRNA expression or activity. HBO therapy reduced lactate formation in both normoglycemic and hyperglycemic rats. These findings support hyperpolarized [1‐^13^C]pyruvate MRI as a novel method for monitoring HBO therapy via the pyruvate to lactate conversion.

## Introduction

A variety of factors contribute to the development of diabetic nephropathy, but intrarenal hypoxia is considered a central factor in multiple forms of chronic kidney disease including diabetic nephropathy (Hansell et al. 2013; Laustsen et al. 2013a; Takiyama and Haneda 2014). Kidney oxygen consumption is increased in diabetes mellitus (Takiyama and Haneda 2014). This increased kidney oxygen consumption is at least partly mediated by increased tubular sodium transport and mitochondrial leak respiration. This process leads to hypoxia due to an imbalance between oxygen consumption and oxygen delivery (Friederich et al. 2008; Hansell et al. 2013). Interestingly, the increased sensitivity to hypoxia in the diabetic kidney may explain the increased prevalence of end‐stage renal disease in diabetic patients living at high altitude (Hochman et al. 2007; Laustsen et al. 2013a). Thus, restoration of intrarenal oxygen availability could be a potential therapeutic target to reduce the risk and rate of diabetic kidney disease.

Hyperbaric oxygen (HBO) therapy is a relatively safe adjunct treatment (Berkovitch et al. 2014; Nikitopoulou and Papalimperi 2015), where patients are repeatedly exposed to elevated oxygen levels for short time periods. In diabetic patients, HBO therapy has been shown to promote healing of chronic foot ulcers and reduce the risk of limb amputation (Kranke et al. 2015). However, the underlying mechanisms of HBO therapy have not been elucidated, although several large‐scale randomized trials have been performed (Margolis et al. 2013; Elraiyah et al. 2016). In this study, we introduced hyperpolarized ^13^C magnetic resonance imaging (MRI) as a new approach for the assessment of endogenous metabolites and their conversion rates in a diabetic rat model. Specifically, this technique requires administration of hyperpolarized ^13^C‐labeled natural occurring derivatives of the metabolism, such as [1‐^13^C]pyruvate (Nelson et al. 2013; Cunningham et al. 2016). Prior to injection, the biomarker has been “hyperpolarized,” which is a process creating a temporary increase of the MRI signal of ^13^C more than 10,000 times compared with normal, nonhyperpolarized ^13^C MRI (Ardenkjaer‐Larsen et al. 2003). A prominent example is [1‐^13^C]pyruvate, which is a substrate for the enzymes alanine aminotransferase (ALT), lactate dehydrogenase (LDH), and pyruvate dehydrogenase (PDH). [1‐^13^C]pyruvate has frequently been used to investigate the metabolic signature of organs in vivo (Kurhanewicz et al. 2011; Rider and Tyler 2013; Laustsen 2016). Pyruvate undergoes metabolic reactions and is converted to metabolic derivatives alanine, bicarbonate, and lactate, which are all detectable by MRI. It has previously been shown that intrarenal pyruvate to lactate conversion is increased in the diabetic kidney (Laustsen et al. 2013b) and that this increase is not affected by insulin treatment (Laustsen et al. 2014). However, chronic antioxidant treatment blunts the diabetes‐induced elevation of this metabolic flux (Laustsen et al. 2017). In this study, we question, if increased oxygen availability following HBO therapy can similarly halt the metabolic derangement seen in the diabetic kidney and second, if hyperpolarized [1‐^13^C]pyruvate MR can assess these changes on the intrarenal energy metabolism.

## Research Design and Methods

### Animal model, experimental design, and induction of diabetes

**Figure 1:**
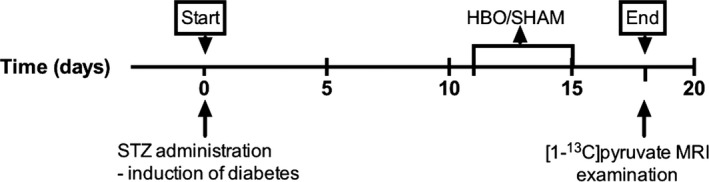
Outline of the experiment. The 5‐day hyperbaric oxygen therapy (HBO) treatment was initiated 11 days after induction of diabetes, and hyperpolarized [1‐^13^C]pyruvate MRI examination was performed 3 days after the end of HBO.

This study was performed under the regulations and guidelines for care and use of laboratory animals and was approved by The Danish Animal Experiments Inspectorate. Twenty‐seven female Wistar rats (Taconic, Ry, Denmark), 8 weeks of age (weight: 250 g), were included in the study. All animals had free access to water and standard chow throughout the study. The rats were housed under a 12:12‐h light:dark cycle, a temperature of 21 ± 2°C, and a humidity of 55 ± 5%. After 1 week of acclimatization, the rats were randomized to one of four groups, consisting of normoglycemic controls and diabetic rats with and without 5 days of HBO treatment. Figure 1 shows the experimental timeline. To differentiate the acute metabolic changes as previously demonstrated (Laustsen et al. 2013a), a wait between the HBO treatment and the MR examination of 3 days was introduced.

Type 1 diabetes mellitus was induced using 55 mg/kg of streptozotocin (STZ; Sigma‐Aldrich, St. Louis, MO) 11 days prior to either HBO therapy or sham therapy. The rats were anesthetized with sevoflurane (3% isoflurane, 2 L/min air) and freshly prepared STZ dissolved in 0.3 mL of 10 mmol/L cold citrate buffer (pH 4.5) was administered intravenously into the tail vein. Sucrose (15 g/L) was added to the drinking water to avoid hypoglycemia following diabetes induction (Tesch and Allen 2007). Blood glucose was measured in blood collected from the tail vein using a Contour‐XT blood glucose meter (Bayer Diabetes Care, Copenhagen, Denmark), and the rats were considered diabetic, if the blood glucose levels exceeded 15 mmol/L at 48 h after STZ administration. Blood glucose was monitored in the diabetic groups 2–3 times per week throughout the study. At the time of euthanasia, both kidneys were removed for biochemical analysis.

### Hyperbaric oxygen therapy

HBO therapy and sham therapy were performed daily for 1 h in a custom‐made pressure chamber (volume 75 L) for five consecutive days. HBO therapy was performed using a pressure of 258 kPa (2.5 × absolute atmospheric pressure) using 100% oxygen. In brief, the rats were placed in the pressure chamber, which was preflushed with oxygen at normobaric pressure for 10 min. Subsequently, the pressure chamber was pressurized at a rate of 30 kPa/min. A similar decompression rate was used following 1 h exposure at 258 kPa. Sham animals underwent a protocol similar to HBO animals; however, sham animals were neither exposed to oxygen nor to pressurization. A gas flow rate of 15 L/min was used whenever animals resided in the pressure chamber, and a CO_2_ scrubber was further used to reduce the CO_2_ level in the pressure chamber.

### Hyperpolarization and MRI examinations

On the day of MRI examination, the animals were anesthetized with sevoflurane (3% sevoflurane and 2 L/min air), and the tail vein was catheterized (G 24) to allow intravenous administration of 1.5 mL hyperpolarized [1‐^13^C]pyruvate. The animal was placed in the MRI system, and the temperature was maintained at 37°C (SA Instruments, Stony Brook, NY). Blood oxygen saturation and respiratory frequency were monitored throughout the experiment using a dedicated MRI‐compatible pulse oximeter and respiration pillow (SA Instruments). MRI and hyperpolarization examinations were performed on a 3T MRI system (GE Healthcare, Waukesha, WI). A dual tuned ^1^H/^13^C quadrature volume transmit/receive coil (GE Healthcare) was used for ^1^H/^13^C excitation/reception. The kidneys were localized by a standard gradient‐echo sequence, and a slice covering both kidneys was shimmed automatically (correction of magnetic field inhomogeneity). A slice‐selective ^13^C IDEAL spiral sequence was used for hyperpolarized [1‐^13^C]pyruvate imaging acquiring images every 5 sec initiated 20 sec after the start of injection. Flip angle = 10°, 11 IDEAL echoes and one initial spectrum per IDEAL encoding, TR/TE/ΔTE = 100 msec/0.9 msec/0.9 msec, FOV = 80 × 80 mm^2^, 5 × 5 mm^2^ acquired resolution, and an axial slice thickness of 15 mm, covering both kidneys.

A volume of 100 *μ*L of 127 mg of [1‐^13^C]pyruvic acid was mixed with 15 mmol/L AH11150 and was inserted in a 5T SPINLab (GE Healthcare, Brøndby, Denmark), in which polarization of more than 40% was achieved, with a final concentration of approximately 100 mmol/L. The sample pH was approximately 7.4, and the temperature prior to injection was approximately 37°C. A volume of 1.5 mL was injected into the tail vein over 10 sec. The transfer time between dissolution and injection was 20 sec on average, and MRI/MR spectroscopy data acquisition was initiated 20 sec after the start of injection.

### Image analyses

^1^H and ^13^C MR DICOM images were transferred to OsiriX (Pixmeo, Geneva, Switzerland) for anatomical overlay and region of interest (ROI) analysis. The metabolite signal was normalized relative to the pyruvate signal.

### LDH activity assay

The LDH activity assay was performed according to the manufacturer's instructions (Sigma‐Aldrich, Brøndby, Denmark). Renal cortical tissue was dissected from the rat kidney and instantly frozen in liquid nitrogen and stored at −80°C. Hereafter, tissue was homogenized in a LDH assay buffer and subsequently, the solution was centrifuged. The supernatant was stored at −80°C. Analysis was performed in 96‐well costar half plates in a PHERAstar FS microplate reader (BMG Labtech, Birkerød, Denmark). Activity measurements were normalized to protein amount in the LDH sample solution. Protein quantification was performed with a BCA protein assay according to the manufacturer's instructions (Fisher Scientific, Roskilde, Denmark).

### RNA extraction and quantitative PCR

Total RNA was isolated from renal cortex using NucleoSpin RNA II mini kit according to the manufacturer's instructions (AH diagnostics, Aarhus, Denmark). RNA was quantified by spectrophotometry and stored at −80°C. cDNA synthesis was performed with RevertAid First strand cDNA synthesis kit (MBI Fermentas, Burlington, Canada). qPCR was performed using Maxima SYBR Green qPCR Master Mix according to the manufacturer's instructions (AH diagnostics, Aarhus, Denmark). Briefly, 100 ng of cDNA was used as template for PCR amplification. Specificity of products was confirmed by melting curve analysis and by gel electrophoresis. Primer sequences used were as follows: *β*‐actin forward 5′‐AGC CAT GTA CGT AGC CAT CC‐3′, reverse 5′‐TGT GGT GGT GAA GCT GTA GC‐3′; LDHA forward 5′‐GCC ATG TAT TCC TTC CCT CA‐3′, reverse 5′‐GCC TCA TTG AAG ACC TGC TC‐3′.

### Statistics

Normality was assessed with quantile–quantile plots. Statistical analysis was performed using GraphPad Prism (GraphPad Software, La Jolla, CA). A two‐tailed *P* < 0.05 was considered statistically significant. Comparisons of animal and kidney weight, endpoint blood glucose, and pyruvate ratios were analyzed by a two‐way ANOVA with a Holm–Sidak post hoc test (two comparisons per family). A linear least squares regression analysis was used to analyze the dependencies between LDH activity and lactate to pyruvate conversion. Blood glucose values exceeding the limits of the measuring equipment were set to 33.3 mmol/L being the upper limit of the apparatus. One animal was excluded from the analysis due to incomplete STZ induction.

## Results

All included rats receiving streptozotocin developed hyperglycemia as confirmed by a blood glucose level higher than 15 mmol/L within 48 h after streptozotocin injection. At the end of the study period, body weight of the diabetic rats was lower than controls, whereas blood glucose levels and kidney weight were higher. A significant difference in response to HBO treatment was found between the diabetics and controls with a 15% lower blood glucose in the diabetic rats and a 12% higher blood glucose level in the control rats (Table 1).

**Figure 2:**
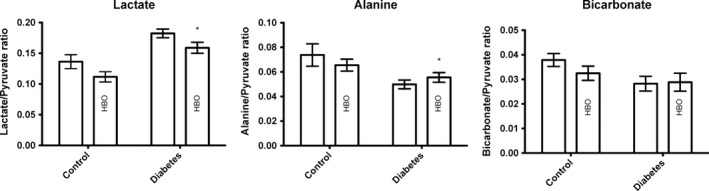
Renal lactate to pyruvate, alanine to pyruvate, and bicarbonate to pyruvate ratios in control and diabetic rats with and without HBO treatment. *P < 0.05 between control and diabetes. (Lactate) *P < *0.0001* for group (between control and diabetes), *P *= 0.02 for treatment (*HBO* treatment groups), and *P = *0.95 for interaction; (Alanine) *P* = 0.004* for group, *P* = 0.80 for treatment, and *P* = 0.19 for interaction; (Bicarbonate) *P* = 0.050 for group, *P* = 0.46 for treatment, and *P* = 0.36 for interaction.

The pyruvate to lactate ratio was significantly higher, whereas the alanine to pyruvate ratio was lower in diabetic rats compared with controls (Fig. 2). HBO treatment of diabetic rats statistically significantly lowered the lactate to pyruvate ratio, but no difference was detected on the alanine to pyruvate ratio.

The diabetic rats showed a statistically significantly lower bicarbonate to pyruvate ratio compared to controls (*P* = 0.05; Fig. 2). No HBO effect was detected on this parameter.

**Figure 3:**
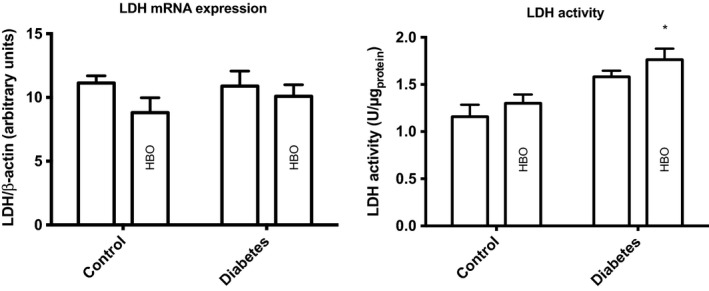
Renal LDH enzyme activity and mRNA expression in controls and diabetes with and without HBO treatment. *P < 0.05 between control and diabetes. (LDH activity) *P* < 0.001 for group (between control and diabetes), *P* = 0.13 for treatment (HBO treatment groups), and *P* = 0.09 for interaction; (LDH expression) *P* = 0.62 for group, *P* = 0.15 for treatment, and *P* = 0.47 for interaction. LDH, lactate dehydrogenase.

**Figure 4:**
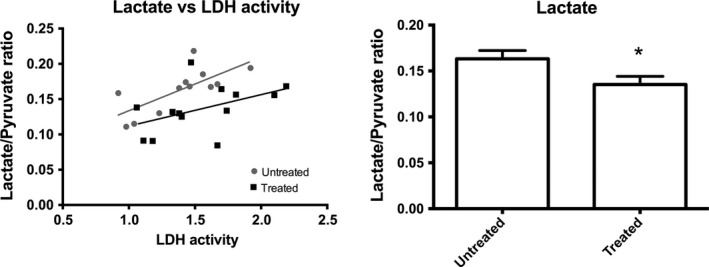
Correlation between LDH and lactate to pyruvate ratio in controls and diabetes with (treated) and without HBO treatment (untreated). A statistically insignificant difference between the two slopes (*P* = 0.39) between the untreated groups, that is, the sum of untreated controls and diabetes animals (*R* ^*2*^ = 0.53, *P* = 0.008), and the treated groups, that is, the sum of the HBO treated controls and the diabetes animals (*R* ^*2*^ = 0.22, *P* = 0.11). The HBO‐treated animals showed a significantly reduced lactate to pyruvate ratio compared with untreated animals, *P* = 0.037. LDH, lactate dehydrogenase. *P < 0.05 between untreated and treated.

**Figure 5:**
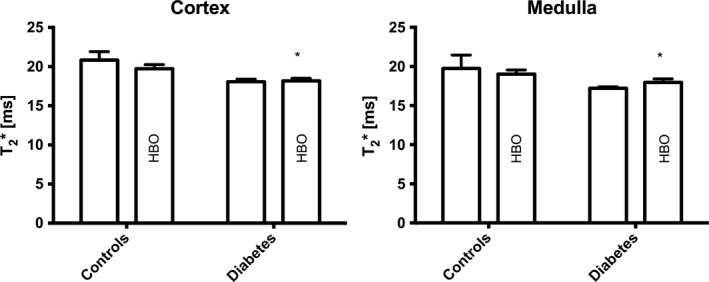
Blood‐oxygen‐level‐dependent magnetic resonance imaging in controls and diabetes with and without HBO treatment. *P < 0.05 between control and diabetes. (Cortex) *P* = 0.0005* for group (between control and diabetes), *P* = 0.35 for treatment (HBO treatment groups), and *P* = 0.25 for interaction; (Medulla) *P* = 0.02* for group, *P* = 0.98 for treatment, and *P *= 0.31 for interaction.

No difference in the LDH mRNA expression was detected between controls and diabetic animals, whereas LDH activity was statistically significantly increased in diabetes (Fig. 3). No HBO effect was detected on the LDH mRNA expression or the LDH activity. Comparing the effect of the in vivo lactate production (lactate to pyruvate ratio) of both controls and diabetic animals with the LDH activity showed a general correlation between the two (*R* ^*2*^ = 0.2, *P* = 0.04), with a lactate lowering effect of HBO treatment (Fig. 4). The blood‐oxygen‐level‐dependent T2* relaxation times in diabetic rats were significantly decreased in both cortex and medulla compared to controls (Fig. 5). No HBO effect was detected on this parameter.

## Discussion

Our results demonstrate that HBO therapy reduced intrarenal lactate in both normoglycemic control and diabetic rats. The reduced lactate formation occurred regardless of little effect of HBO on the LDH mRNA expression and activity found with biochemical assays. HBO therapy significantly reduced the blood glucose levels in the diabetic animals, although the diabetic animals receiving HBO still had pronounced hyperglycemia in the range previously demonstrated to induce significantly elevated intrarenal lactate formation (Laustsen et al. 2013a,b, 2014; von Morze et al. 2016). Similarly, reduced fasting blood glucose during HBO is known from human studies of type 1 and type 2 diabetes and in diabetic rats (Yasuda et al. 2006; Wilkinson et al. 2012; Stevens et al. 2015). A similar decrease in blood glucose was observed in the present study.

In contrary to our findings, Matsunami et al. (2008) reported increased blood glucose levels in a similar animal model of experimental type 1 diabetes, and they found a reduction in the insulin‐producing *β*‐cells in diabetic rats receiving HBO therapy compared with a control group. These differences might be attributed to different levels of diabetes. Another study reported unaltered blood glucose levels when exposing healthy rats to HBO up to 4 ATA (Eynan et al. 2015). In that study, pressure above 4 ATA was reported to increase blood glucose levels; therefore, it was suggested that HBO therapy induces glycogen depletion and insulin resistance mediated by the sympathetic nervous system. In the present study, we waited for 3 days after the end of HBO therapy before performing hyperpolarized [1‐^13^C]‐pyruvate MRI assessment since we did not want to examine the acute effects induced by rapid alterations in pressures and stress, which might conceal potential long‐term effects of HBO. The above‐mentioned studies (Matsunami et al. 2008; Eynan et al. 2015) used a very short time interval between HBO therapy and the subsequent examination of its effects. The difference between the acute and delayed examinations suggests a potentially delayed conditioning effect of the HBO therapy, which might differentiate the acute and long‐term HBO effects. Interestingly, Klemetti et al. (2005) have shown a positive effect on blood supply following HBO therapy in rats undergoing mandibular surgery, up to 2 weeks after the treatment.

The positive correlation between LDH activity and the lactate to pyruvate ratio indicates that the LDH flux is directed toward lactate production (increased glycolysis), suggesting that the lactate pool size is less determined by the gluconeogenesis (lactate utilization) following HBO treatment (Lewis et al. 2016; von Morze et al. 2016). These findings demonstrate an interesting phenomenon with a preconditioned LDH expression/LDH activity (tendency toward decreased LDH expression and increased LDH activity in the HBO‐treated animals), potentially driving the increased lactate utilization in both control and diabetic animals (lower pyruvate to lactate ratio following HBO therapy).

The pyruvate to bicarbonate conversion is an indicator of the mitochondrial energy production and thereby an indicator of pyruvate dehydrogenase activity (Golman et al. 2008; Laustsen et al. 2013b). In our study, we found that the control group overall had the highest pyruvate dehydrogenase activity, whereas the diabetic groups had a tendency toward a lower pyruvate to bicarbonate ratio indicating a decrease in the pyruvate dehydrogenase activity. The increased lactate level can be attributed to the polyol pathway (Palm et al. 2004); however, increased anaerobic metabolism due to renal hypoxia will also contribute to this increase. Interestingly, Lou et al. (2007) found that rats exposed to brain ischemia and subsequently treated with HBO therapy had a smaller infarct volume than control groups due to normalized glucose utilization in the ischemic zone in the HBO‐treated animals. If the same physiology can be applied to hypoxia in the kidneys, this explains our observed tendency toward increased lactate utilization in the diabetic HBO‐treated group as compared with the untreated diabetic group, that is, HBO therapy seems to increase the metabolic demand.

We speculate that the beneficial effects might be more pronounced, when blood glucose is chronically elevated, as seen in poorly controlled diabetes, where increased lactate formation might contribute to the development of diabetic nephropathy.

It is important to note that the current study was performed on female rats and the STZ‐induced type 1 diabetes model. It is currently unknown if gender or other models of type 1 or type 2 diabetes‐specific alterations are associated with a similar positive response to HBO therapy.

To the best of our knowledge, this study is the first to show the effect of HBO therapy on the intrarenal metabolism in healthy and diabetic rats. Compared with previous studies using dialysis, blood and urine samples, invasive probes, and in vitro measurements, this study provides a noninvasive in vivo assessment of the kidney metabolism. Using this method, we found that HBO can alter the metabolism in both healthy and primarily diabetic rat kidneys. We found a decrease in the blood glucose level after HBO in the diabetic rats. This decrease is also found in the human type 2 diabetes mellitus after HBO and is attributed to improved peripheral insulin sensitivity (Wilkinson et al. 2012). The physiological explanation for this change has yet to be elucidated for type 1 diabetes mellitus, but could be the same as for type 2 diabetes mellitus.

In conclusion, HBO therapy reduces intrarenal lactate production without affecting either LDH mRNA expression or LDH activity. A similar lower renal lactate production has recently been demonstrated in response to antioxidant treatment (Laustsen et al. 2017). HBO therapy seems to reduce blood glucose levels in diabetic rats, and the treatment decreases the metabolism in the healthy rat kidney and attenuates the derangement of metabolism in the diabetic kidney. This novel finding indicates that HBO therapy may be a clinically useful adjuvant therapy in diabetes patients at risk of developing renal disease. Further studies are needed to clarify the exact mechanisms.

### Summary and significance

Intrarenal hypoxia has been shown to be an important mediator in renal disease and in diabetic nephropathy in particular. Hyperbaric oxygen therapy is an appealing treatment modality, albeit metabolic effects have so far been challenging to investigate. The present study demonstrated that HBO therapy improvements in insulinopenic rats can be monitored via the intrarenal alterations in the pyruvate to lactate conversion.

## Conflict of Interest

There are no conflicts of interest to report in relation to this work.
